# Exploring Feedstock Recycling in Liquid-Phase-Exfoliated Nanosheets

**DOI:** 10.1021/acssuschemeng.4c05845

**Published:** 2024-09-18

**Authors:** Jacob Brown, Jason Stafford

**Affiliations:** School of Engineering, University of Birmingham, Birmingham B15 2TT, United Kingdom

**Keywords:** Nanomaterial, Liquid Phase Exfoliation, MoS_2_, Solvent Recycling, Green Solvent, Alcohol Cosolvent

## Abstract

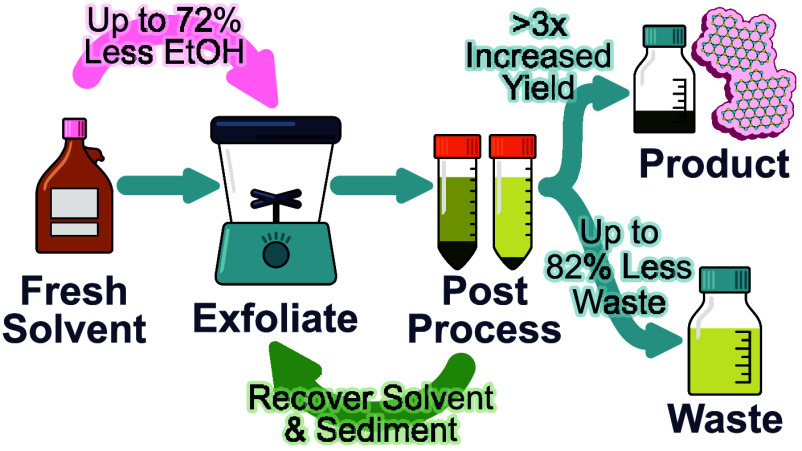

Industrial scale-up of two-dimensional (2D) nanomaterial production is essential if these novel materials are to prove themselves commercially viable for applications ranging from energy conversion and storage to optoelectronic devices and clean water. There are several techniques to produce 2D materials, and liquid phase exfoliation (LPE) is one that has emerged as the most widespread across laboratory, pilot, and industrial production scales. One of the main issues faced in the scale-up of such techniques is the low yield (typically 0.5–5 wt %), resulting in high levels of wasted feedstock material. Similarly, every 10 kg of product can create 1000 L or more of solvent waste, many of which are highly toxic to the environment (e.g., NMP). Using MoS_2_ as a prototypical 2D material, this work demonstrates a sustainable approach to recover and reuse unexfoliated precursor material and the exfoliation solvent to reduce waste by up to 82% and solvent requirement by up to 72%. Material production efficiency benefits were also achieved, with over 3-fold increase in product yield and energy usage reduced by up to 12%. The approach can be easily scaled and immediately implemented using existing infrastructure, and a pathway for industrial implementation has been outlined to support this.

## Abstract 2

Solvent used in nanomaterial synthesis is recycled to reduce hazardous waste production and improve product yields.

## Introduction

Liquid phase exfoliation (LPE) is a top-down nanomaterial synthesis, where a layered precursor material such as graphite, molybdenum disulfide (MoS_2_), or tungsten disulfide (WS_2_) is dispersed in a liquid dispersant such as a solvent or surfactant solution and broken down to nanoscale particles, typically using high-shear mixing or sonication.^1,2^ These methods have great potential for industrial scale-up,^2−4^ with pilot processes having already been implemented in the past decade.^5^ One issue, however, not just with LPE but all nanomaterial synthesis, is low yield and a dependency on large volumes of solvent during material synthesis and chemical workup stages resulting in substantial quantities of hazardous waste.

Maximizing materials efficiency is crucial environmentally and economically, especially for technologically “critical” materials such as graphite, but also other abundant layered materials such as MoS_2_ and the solvents used. While recycled feedstocks from other end-of-life applications (e.g., spent battery anodes)^6,7^ is a promising start to reducing environmental impact and addressing other emerging waste management challenges, it is not a solution for poor process efficiency, and raw material feedstock sources will remain a crucial part of the manufacturing economy.

Several papers discuss the recovery and reuse of unexfoliated precursor material.^8−10^ This can involve collecting and drying residual material, an energy and time intensive process with possible health and safety risks when handling dry nanomaterial powders. In other instances, residual material is collected via centrifugation and redispersed in fresh solvent.^5,11^ Single, high yield processes with 0.5–5 wt % have been improved up to 10–50 wt % in methods using precursor recycling;^9−11^ although values vary significantly between sources. These ideas improve precursor utilization; however, the large volumes of nanomaterial-contaminated waste solvent are not mitigated.

Excessive solvent waste is often exacerbated by the use of traditional toxic solvents such as dimethylformamide (DMF) and *N*-methylpyrrolidone (NMP).^12−14^ Even “safer” alternatives such as dispersant-assisted exfoliation,^1^ e.g., using surfactants, can be toxic to aquatic ecosystems^15^ or require additional postprocessing to remove excess dispersant which may introduce undesirable properties. Many researchers have investigated the use of less harmful “green solvents”, including alcohol–water cosolvents (e.g., ethanol (EtOH)/DeI, isopropyl alcohol (IPA)/DeI), biobased alternatives to NMP (such as cyrene), and novel products such as PolarClean and Iris which are nontoxic, produce yields comparable to NMP, and advantageous nanosheet aspect ratios, but tend to have high boiling points (>200 °C), increased viscosity compared with alcohol–water cosolvents, and higher costs.^9,11,16−21^

Suitable dispersant selection is crucial to the production of nanomaterials via LPE as factors such as the solubility parameters, and viscosity can have drastic effects on the synthesis efficiency, postprocessing, and morphology of the final product in addition to the discussed environmental and health related concerns.^2,19,22,23^

As well as using greener solvents, reducing waste volume is crucial for production scale-up. Currently, many LPE processes require over 1000 L of solvent to produce 10 kg of the nanomaterial product. Optimizing production parameters such as batch or continuous flow designs, shear rates, and mixing efficiency can go some way to improving this. As an additional or alternative approach, we can minimize the production of waste solvent by reusing and recycling. Wrasman et al.^24^ used an antisolvent to redisperse nanoparticles, allowing the initial solvent to be reused. Ionic liquids offer alternative solvents that may provide recycling routes;^25,26^ however, these dispersants can be expensive and have high viscosity presenting potential challenges for LPE.^2,22^ Although the use of distillation processes to recover and reuse solvents has been demonstrated in other production processes such as polystyrene nanomaterials,^27^ the authors were unable to find examples of investigations on LPE synthesis where the *solvent* was recycled.

To overcome this and develop sustainable 2D material synthesis strategies that can be extended to industrial-scale manufacturing, this study focuses on exploring feedstock recycling using MoS_2_ as the model material. Molybdenum disulfide was chosen as it is one of several promising semiconducting materials (transition metal dichalcogenides^28^) and presents edge and quantum confinement effects^29^ that allow for an efficient examination on how recycled feedstocks impacts nanosheet yield, size, and thickness statistics using optical spectra. The method demonstrated in the current work uses liquid cascade centrifugation (LCC) postprocessing^30^ to reuse solvent several times, increasing cumulative product yield and reducing waste production significantly. We then explore additional recycling opportunities through distillation and compare the production output, energy, and material resource efficiency to understand the combined sustainability and performance gains that can be achieved using these methods. Given that mechanical exfoliation is an agnostic approach for synthesizing van der Waals materials in liquids, the method outlined is applicable to any polar solvent/layered material combination.^16,31^

## Experimental Section

### Materials and Equipment

Ethyl alcohol (Fisher Scientific, 10437341), deionized water (5 MΩm), and molybdenum(IV) sulfide (MoS_2_ powder, Sigma-Aldrich, 69860) were used. MoS_2_ required a further purification step, detailed below. An AMZChef 2 kW kitchen blender modified to allow speed control with an arduino controlled relay to give timing control was used to synthesize nanomaterial dispersions. The procedures required to perform these modifications are provided in our “OpenLPE” open-source repositories.^32^ The device was operated at a fixed impeller speed of 11,000 rpm resulting in an average shear rate ∼10^4^ s^–1^ within each 400 mL batch. Full details of the synthesis procedure can be found in the Supporting Information (SI). A power metering socket (RS Pro 178-5370) was used to measure the power used by the blender for synthesis, centrifuge for postprocessing, and rotary evaporator for distillation.

### Precursor Purification

As-received MoS_2_ powder contains unidentified soluble impurities which destabilize nanomaterial dispersions.^33^ These impurities may be soluble Mo, S species, formed by dissolution of an oxidized surface layer on the MoS_2_;^34,35^ however, an exact identification and quantification falls outside of the scope of this work. Importantly, the removal of these impurities is an essential step for a successful synthesis. We find, in agreement with Griffin et al., who performed synthesis using ultrasonication,^33^ no measurable MoS_2_ yield in the absence of a cleaning process. Batches of MoS_2_ powder were briefly shear-exfoliated (≈ 10 min total exfoliation time) in DeI water and washed through a “qualitative” paper filter with additional DeI. The resulting powder was dried at ≈60 °C and stored in an airtight container. This approach confirms that shear-exfoliation processes can be used to perform both the preclean and nanosheet synthesis steps, a beneficial characteristic when considering process scalability.

### Synthesis and Recycling Procedure

**Figure 1:**
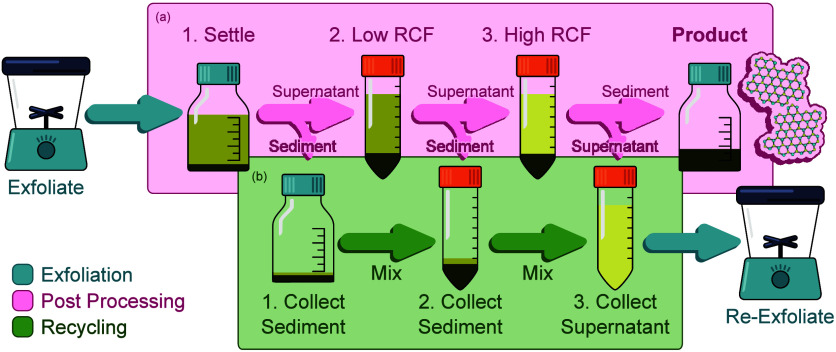
Schematic showing the postprocessing (a) with settling (1) and two stage LCC process (2, 3), indicating the points at which product and “waste” are collected for recycling (b).

In brief (additional methodology details can be found in the SI), precleaned MoS_2_ powder was added to 400 mL of EtOH/DeI cosolvent (50:50 volume ratio) at an initial concentration of *C*_*i*_ = 30 g·L^–1^. This starting concentration was selected as it is an intermediate value from what has typically been used in LPE studies.^5,8,36^ An examination on the effect of *C*_*i*_ on MoS_2_ yield was conducted, and similar trends were observed to previous literature (see SI). After exfoliating the cleaned precursor to synthesize MoS_2_ nanosheets, mixtures were left to settle for ∼24 h (Figure 1a, step 1) before pipetting the supernatant off to reduce the sediment volume prior to centrifugation. The remaining sediment slurry was set aside and retained for subsequent recycling steps. A liquid cascade centrifugation (LCC)^30^ protocol, illustrated in Figure 1, was developed to collect a specific nanomaterial size fraction from a polydisperse sample. Extended details can be found in the SI. For subsequent synthesis iterations, unexfoliated material collected during LCC (Figure 1b, step 2) was combined with the previously retained sediment slurry. This is topped up to ≈400 mL with recovered EtOH/DeI cosolvent, and the exfoliation procedure is repeated, as illustrated schematically in Figure 1.

Two additional variations on this *Recycle* methodology were performed. The first is much the same as previously described but redisperses the unexfoliated sediment (Figure 1b, steps 1 and 2) with fresh EtOH/DeI rather than the recovered supernatant, as in Figure 1b, step 3. In the second variation, precursor material was continuously exfoliated for a total of 90 min, using the same 1 min “on” and 2 min “off” procedure as the main experimental method. This approach of continuous exfoliation without solvent recycling is the typical method performed in the scientific literature and at industrial manufacturing scales. To ensure the same cosolvent mix was maintained across experiments, EtOH concentration was monitored and adjusted as necessary (see SI).

### Characterization

The concentrations of MoS_2_ nanosheet dispersions were determined using UV–vis spectroscopy (PerkinElmer Lambda 365), the Lambert–Beer relationship (eq 1), and an experimentally measured extinction coefficient, ε_345_ of 44.40 mL mg^–1^ cm^–1^. From the extinction spectra obtained, the average nanosheet layer number and length were calculated based on metrics proposed by Backes et al. in eqs 2 and 3.^29^ To confirm the nanosheet size and examine if recycling introduced any variations in morphology, scanning electron microscopy (SEM) was performed on exfoliated samples. As-prepared nanomaterial dispersions were drop cast on a glass slide and sputter coated (Quorum Q150T ES Plus) with ≈8 nm thick gold coating for SEM imaging (Apreo 2S HiVac).
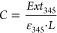
1
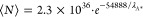
2
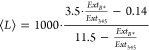
3

## Results and Discussion

### Impact of Recycling on Synthesis

**Figure 2:**
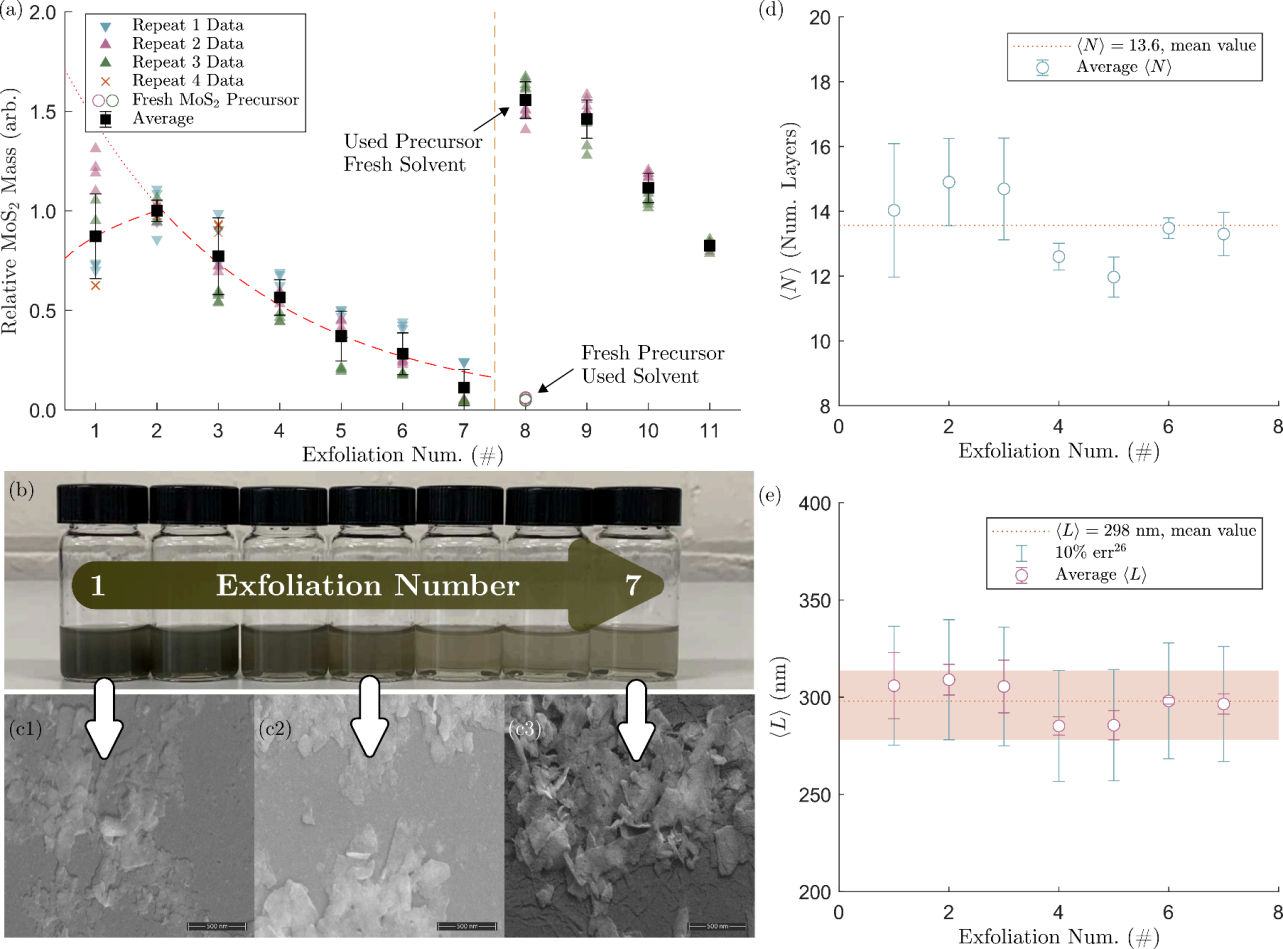
(a) Effect of solvent recycling on the mass yield of MoS_2_ nanosheets. Data presented as relative mass, which describes the nanomaterial mass relative to the maximum yield observed at the second recycle stage. Experimental repeats are represented by different marker colors. Different precursor cleaning batches are represented by marker shape (Δ,∇, × ). Point one on the exfoliation number axis is the initial synthesis using virgin feedstock materials, subsequent points follow the recycling methodology. Yellow dashed line indicates the point at which fresh solvent/precursor material was introduced. (b) Images of MoS_2_ dispersions from repeated exfoliations 1–7, diluted 5:1 to highlight concentration change. (c) SEM images of MoS_2_ nanosheets from exfoliations 1, 4, and 7. Effect of solvent recycling on (d) average number of atomic layers (error bars indicate standard deviation of measurement repeats). (e) Average nanosheet length (blue (outer) error bars indicate 10% metric uncertainty;^29^ pink (inner) error bars indicate standard deviation of measurement repeats). The dotted lines indicate averages of all exfoliation numbers.

The effect of the number of recycles on MoS_2_ nanosheet production is shown in Figure 2a, with the data normalized by the maximum mass observed at the second recycle stage. An exponential decrease in MoS_2_ mass per synthesis starts at the second recycle, with the initial synthesis using virgin materials showing lower production output relative to this. It was initially hypothesized that this reduction in product mass may be due to a decrease in the effective starting concentration (*C*_*i*_) because of the potential loss of MoS_2_ when transferring between containers and removing product. To test this, the sediment from iteration 7 was exfoliated with fresh solvent, and the recovered solvent from iteration 7 was used to exfoliate the fresh MoS_2_ precursor. There is a drastic difference in yield between these two methods (Figure 2a, point 8 on the exfoliation number axis), with fresh solvent/recovered precursor producing a significant yield, even greater than the initial yield. On the contrary, using the recovered solvent with fresh MoS_2_ precursor produced a low yield, only marginally increased from the previous iterations. This confirms that the reduction in yield is due to solvent effects and likely from an increase in the concentration of ionic impurities. The full optical extinction spectra used to extract nanosheet concentration and morphological statistics for the various repeats are provided in Figure S1.

The increase in yield seen between the first and second iterations is thought to be due to one or a combination of three mechanisms:

1. Recovered solvent has trace nanomaterial concentration, providing a higher baseline to start with.
2. Concentration of ionic impurities in the solvent increases with exfoliation, thus influencing the zeta potential^37^ and possibly improving nanomaterial stability up to a point before becoming detrimental.
3. Precursor material recovered from the first iteration may have a smaller particle size/prefractures/surface preconditioning that reduces exfoliation energy requirements. This is partly supported by the enhancements observed when exfoliating recovered precursor in fresh solvent (Figure 2a, point 8).

Point 2 is likely to have the most dominant effect and is also proposed to be responsible for the variability observed between repeats (Figure S2). There are clear offsets between the initial masses (*M*_*i*_) achieved for different repeats, likely due to the poor repeatability of the precursor cleaning efficacy and differences in impurities between precursor product batches. A differing initial impurity concentration affects the initial yield, and progression of impurity concentration over the subsequent recycling iterations.

Recycling was found to have a negligible effect on the nanosheet morphology (Figure 2d, e). Given the sensitivity of 2D material properties on length and thickness characteristics, this is a beneficial outcome that supports the use of recycling in applications. MoS_2_ sheet thicknesses achieved are larger than would typically be desirable (⟨*N*⟩ < 10),^38^ due to an unoptimized LCC procedure. However, the intention of this work is to demonstrate the efficacy of facile solvent reuse. Similarly, optical extinction metrics from Backes et al. are accurate for 70 < *L* < 350 nm (±10%), and *N* < 10.^29^ Lengths are well within this range (Figure 2e); however, the number of layers is >10 (Figure 2d). Although this is slightly outside the bounds of the empirical correlation, what we can determine is that there are no clear changes or trends to nanosheet size at each recycle iteration; a straight line can be drawn across the data in Figure 2e, within the 10% uncertainty bars between 278.1 and 313.7 nm and an average of 298.0 nm. Scanning electron microscopy images were obtained for different recycle iterations, confirming the consistent nanosheet dimensions observed using the optical extinction metrics (Figure 2c).

### Sustainability

#### Yield and Waste Reduction

**Figure 3:**
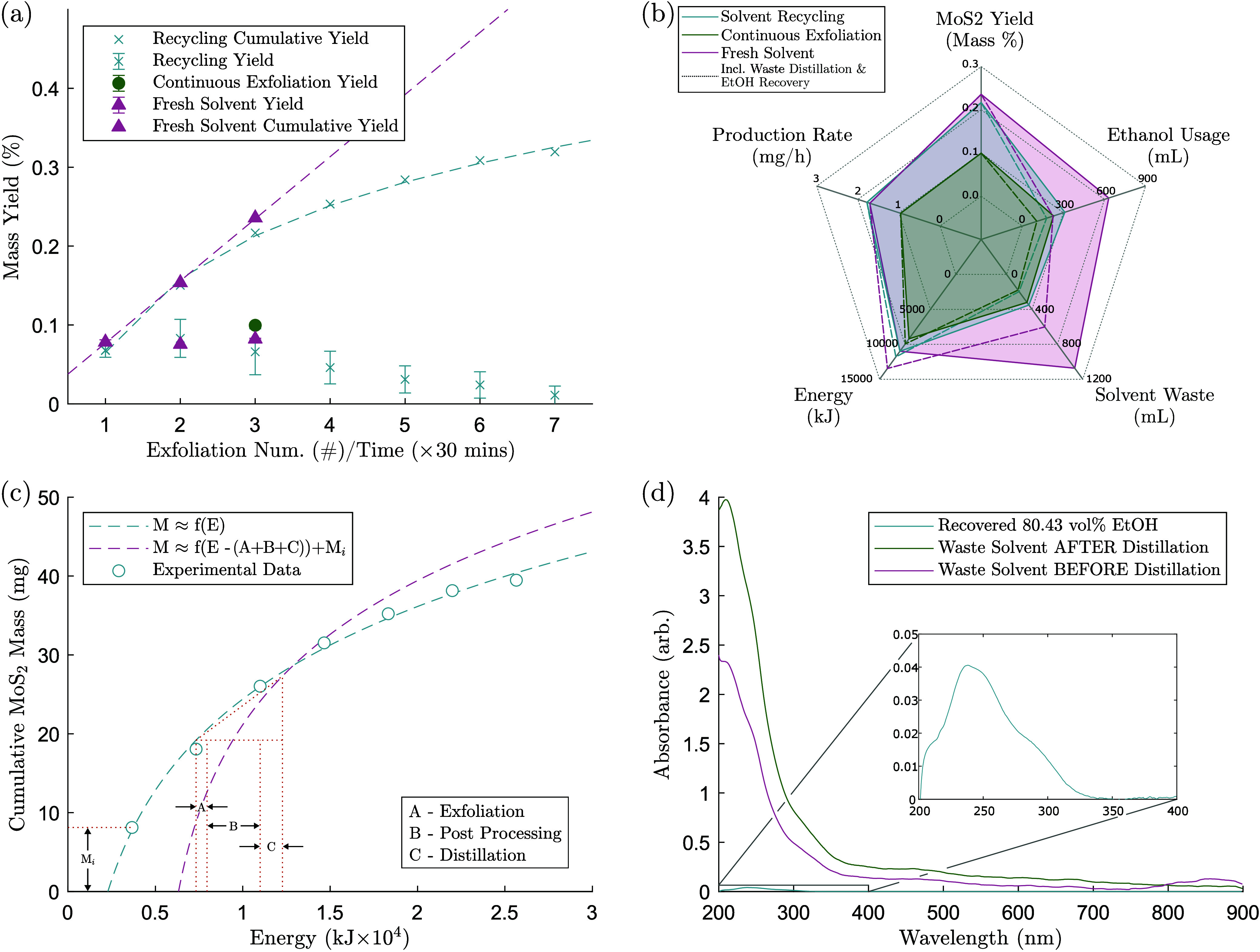
(a) Yield of MoS_2_ for the three different synthesis procedures. Cumulative yield is ∑_*i* = 1_^*n*^*Y*_*i*_, where *Y*_*i*_ is the yield of each exfoliation, up to the exfoliation number, *n*. (b) Comparisons between yield, production rate (based on exfoliation and postprocess time), ethanol usage, energy usage, and waste volume. Dashed lines indicate the results when including the effects of recovering ethanol from waste solvent via distillation. (c) Cumulative mass against energy requirements for main recycling procedure, described in Figure 1, used to determine when it is most energy efficient to distill the waste solvent. (d) Effect of distillation on the absorbance spectra of solvent waste and recovered ethanol.

Key measures of the performance of the synthesis processes are yield and cumulative yield, both of which are shown in Figure 3a for three scenarios of (i) continuous exfoliation without recycling, (ii) recycling the precursor only and using fresh solvent, and (iii) recycling precursor and solvent feedstocks. The yield data present the performance of the synthesis at each recycling interval or exfoliation time, whereas the cumulative yield is a moving sum of the yield. Yields have been improved from an average 0.07 ± 0.01% in the initial “virgin” synthesis to a cumulative 0.32 ± 0.16% after 7 exfoliation cycles, an ∼4.6× increase, with an average of 10.2 mL additional EtOH used at each iteration to adjust the solvent EtOH concentration and 27.3 and 29.0 mL EtOH/DeI cosolvent to recover excess sediment from the exfoliation vessel and low RCF centrifuge tubes, respectively. The ∼29.0 mL to recover material from the exfoliation vessel is necessary for recycling and virgin syntheses. When only the precursor is recycled and fresh solvent is used, this achieves the highest cumulative yield. However, the performance when using the fully recycled feedstocks is remarkably close after 3 iterations. This difference in performance ultimately increases as the solvent effects dominate with increasing recycling intervals, which suggests that an optimal recycling process that maximizes yield and minimizes waste (and energy usage) is possible and is explored further in the next section. Despite this, we clearly demonstrate that the solvent recycling principle is effective, and it is expected that these principles and trends should hold under varying operating conditions. Significantly, the yield achieved after 90 min of continuous exfoliation without recycling was less than 50% of that which can be obtained through feedstock recycling, emphasizing that this sustainability approach for waste reduction can simultaneously improve material conversion. Considering that current laboratory and industrial manufacturing approaches for synthesizing graphene, MoS_2_, and other 2D materials are operating on a continuous exfoliation principle, this finding suggests that environmental, process, and economic gains can be achieved by implementing this feedstock recycling principle.

#### Distillation

Although the benefits of feedstock recycling using the proposed postprocessing procedure illustrated in Figure 1 are evident, there are two limitations. First, the continued degradation in solvent performance after each iteration (Figure 2a) will necessitate its replacement and a reintroduction of fresh solvent to retain reasonable synthesis yields again. Second, the solvent, although having been reused many times, will ultimately require disposal. To address these issues, an experiment into the recovery of ethanol from the waste solvent via distillation was performed. Here, 360 ± 5 mL of waste EtOH/DeI, measured at 45.51 vol %, was distilled using a rotary evaporator (BUCHI Rotovapor R-100). A total of 0.229 kWh (824.4 kJ) was used (inclusive of heating bath, rotation, vacuum pump, coolant pump) to recover 160 ± 2 mL of 80 vol % EtOH. This corresponds to 128 mL of EtOH equivalent once diluted back to the 50 vol % cosolvent ratio and thus 6.41 kJ.mL_EtOH^–1^_. As well as recovering solvent, this leads to a 44% reduction in the volume of solvent waste. Clearly these numbers are specific to our particular lab scale setup and are subject to change when scaled for industry. This experiment was also not optimized, as it is merely a proof of concept. However, distillation is a process that is operated at scale in industry, and it is likely the results are comparable and the method transferable to other lab scale setups which regularly use rotary evaporator equipment to perform chemical separation and purification.

The UV–vis-nIR absorbance spectra of the recovered 80 vol % EtOH shows some evidence of impurities (Figure 3d), but at a significantly reduced level compared with those in the waste solvent (∼60× lower), this reduction would be compounded by diluting back to 50 vol % EtOH. It is difficult to quantify the reduction due to the impurities being unidentified and there being a clear change in the prominence of the various peaks; all three curves show peaks at ∼210, 238, and 249 nm as well as a broad background peak, with the ∼210 nm peak being dominant in the waste solvent and the ∼238 nm peak dominant in the distillate. Normalized absorbance spectra can be seen in Figure S4 for comparison. MoS_2_ is marginally visible in the waste solvent curves (slight humps can be seen at ∼450 and ∼675 nm), but these are dwarfed by the impurity peaks and therefore negligible in comparison.

It is thought that such a significant reduction in the residual impurities concentration would allow the recovered solvent to perform no worse than that of virgin solvent and potentially result in enhanced yields due to the possible ionic stabilization effects discussed in the previous section. Mitigating impurities completely is a significant challenge due to them being a product of the precursor material, which most likely will also have some preoxidation during preparation and storage. It has been shown that MoS_2_ forms an oxidized surface layer which dissolves in water, releasing the Mo and S ionic species that destabilize the dispersion. Deoxygenated aqueous environments have been shown to substantially reduce the dissolution kinetics,^35^ and therefore, this approach might be used to reduce the concentration of ionic impurities formed. If a more complete removal of impurities from the recovered solvent is required, one option may be to combine distillation and ion-exchange filtration, although this would require further research to validate. Provided the recovered ethanol performs “as new” it would seem the suitable time to distill the waste solvent and recover the EtOH would be when the energy required to recover the solvent is exceeded by the energy required to increase the cumulative product mass by the initial synthesis yield, *M*_*i*_ = 8.12 mg. This relationship between the MoS_2_ mass and energy input is shown in Figure 3c. By plotting product mass as a function of energy, and fitting a power law, we can offset this curve in *x* by the energy required per synthesis (3664.8 kJ – (*A* + *B*)) and the energy to distill enough solvent for one iteration (6.41 kJ.mL_EtOH_^–1^ × 200 mL = 1281.2 kJ – (*C*)), and in *y* by *M*_*i*_; i.e., *f*(*x* – 3664.8 – 1281.2) + 8.12, as in Figure 3c. The intersection of these curves, at ≈3.5 iterations, is the point at which it is energy efficient to distill the waste solvent. If the production rate were to hold greater significance than energy consumption, a similar calculation might be performed with time substituted for energy.

A holistic analysis was used to compare the various components of the LPE synthesis process, postprocessing approach, and distillation step with respect to production performance (2D material conversion), energy usage, and waste production. Three recycling iterations were selected for experiments to analyze the relative merits of complete feedstock recycling, fresh solvent usage, and running continuous exfoliation without recycling. A spider plot of the results can be seen in Figure 3b. Over this operation window, by simply switching from fresh solvent at each iteration to reusing the solvent, we observe a 51% reduction in the required EtOH and a 67% reduction in the volume of waste produced. When we factor in distillation of the waste for both methods, EtOH use is 21% lower for recycling than fresh solvent due to the increased amount of EtOH recoverable from the larger waste volume of the fresh solvent scheme, and we can see up to 82% reduction in solvent waste and 72% reduction in EtOH use when comparing solvent recycling with EtOH recovery via distillation with using fresh solvent at each iteration. Without distillation both methods use the same energy, but due to the increased waste volume, when distillation is considered, the fresh solvent method uses 14% more energy than the recycling method. On top of these obvious benefits, a small decrease in yield from 0.236% for fresh solvent to 0.217% for the recycling scheme was observed, although this may be due to the discrepancies in yield attributed to the presence of ionic impurities. When comparing the values from the same precursor cleaning batch, which can be expected to produce more repeatable results, the fresh solvent yield is 0.244%, which is within the combined standard deviation for yield measurements (±0.015%).

Exfoliating “continuously” for the same cumulative exfoliation time (90 min) is the least effective in all aspects; a much lower yield (0.100%) is achieved. While this method uses marginally less EtOH and produces marginally less solvent waste than the recycling method, the volumes per milligram of product are all worse than the other methods. Again, this finding emphasizes the advantages to sustainable manufacturing of 2D materials by implementing feedstock recycling, alongside optimizing typical synthesis parameters such as exfoliation time, synthesis volume, temperature, etc. For the latter, this also depends on the LPE technique (e.g., shear-mixing, ultrasonication). It has been shown previously that shear-mixing can be more efficient than sonication, especially when scaling to larger volumes of ∼100 L.^5^ Despite the potential differences in efficiency between exfoliation approaches, the feedstock recycling method demonstrated in this work can be applied to all LPE synthesis techniques.

### Scale-Up

**Figure 4:**
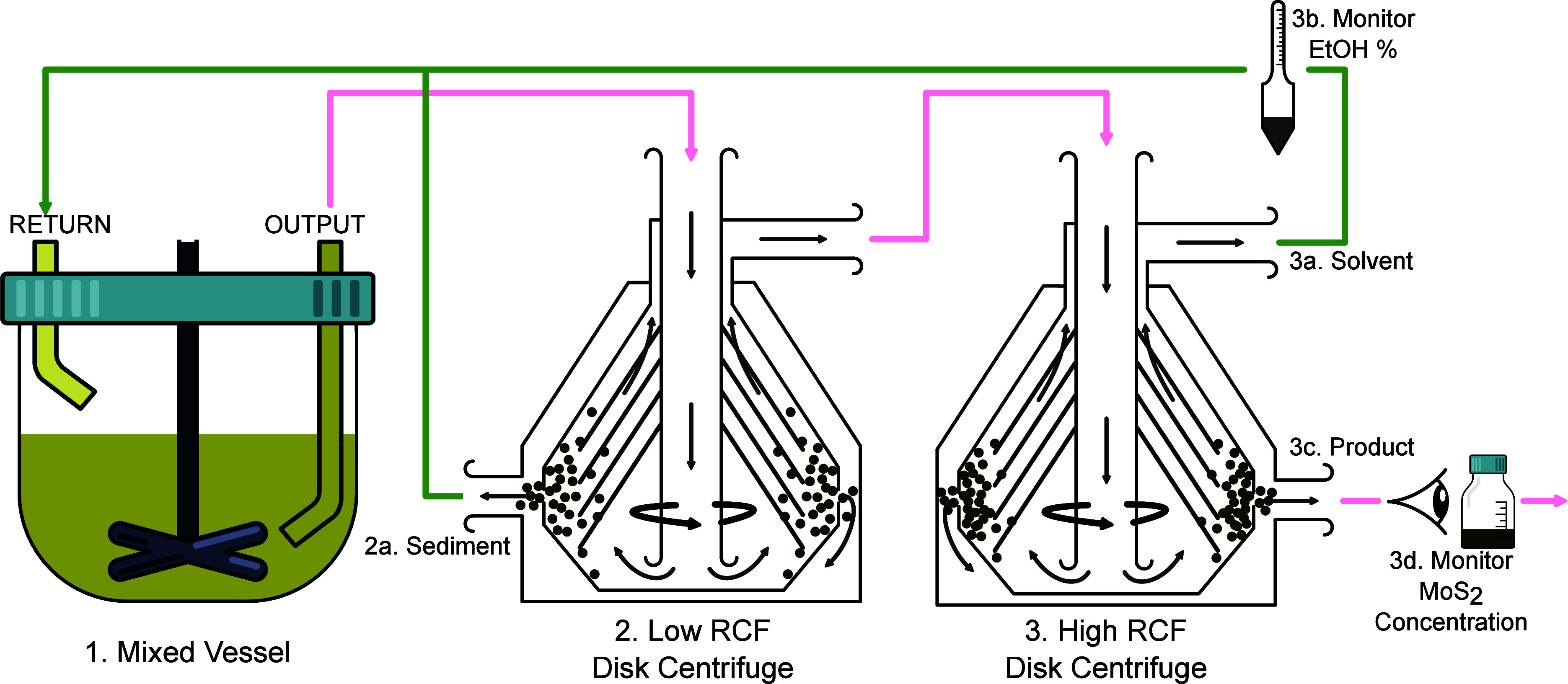
Proposed schematic for an industrial constant/batch production implementation utilizing two continuous flow disk centrifuges which return “waste” material back to the exfoliation vessel. Ethanol concentration should be monitored (3b), e.g., by hydrometer measurements to determine the density, and adjusted if necessary. MoS_2_ and impurity concentration in the product should be monitored (3d).

Our investigation has been conducted on a laboratory scale, which necessitates considerations for industrial implementation. The concepts and principles outlined in this work have been proven for technologies that have been scaled up previously across different manufacturing industries. For example, high-shear LPE is in use for large-scale production of few-layer graphene. Distillation processes are used in numerous chemical manufacturing industries, and sedimentation-based centrifugation technologies are also utilized for material separations. To support the translation of these findings into large-scale manufacturing, a possible implementation is illustrated schematically in Figure 4, in which the material recycling principles demonstrated here might be used to increase product yield and reduce material costs and environmental impact. Material can be exfoliated continuously in a large mixed vessel with a continuous outflow/return. It is important to highlight that any high-shear or mechanochemical exfoliation process could be used here, and this mixing vessel arrangement is what most closely reflects the laboratory equipment used for this study. Two constant flow disk-centrifuges are used for the LCC processing and output sediment (Figure 4: 2a), solvent (Figure 4: 3a), and product material (Figure 4: 3c). Recovered solvent EtOH concentration should be monitored (Figure 4: 3b) and adjusted as necessary, before being combined with the collected sediment and returned to the exfoliation vessel. With proper cooling, it is thought that solvent evaporation should be minimal, and EtOH concentration adjustment may not be necessary. A final solvent distillation step would then be performed when the energy requirements are found to be favorable to production efficiency (e.g., as performed in Figure 3c) by connecting the solvent outlet (Figure 4: 3a) to a distillation process plant and feeding the distillate back to the return of the mixed vessel. The primary drawback to this type of system would be ensuring a consistent and sufficient removal of ionic impurities during the precursor cleaning stage; it may also be beneficial to monitor impurities concentration in the solvent during synthesis, although it may be nontrivial to obtain accurate quantification using spectrographic means due to convolution with the trace MoS_2_ present in the solvent. Possible workarounds may include at-line measurements that allow MoS_2_ to be removed by filtration or an alternative proxy-metric such as measuring solvent conductivity. Monitoring the MoS_2_ concentration in product outflow (Figure 4: 3c) may be challenging to implement in a constant-flow inline way when using spectrographic metrics, as used in this work. Implementing an at-line solution would be feasible but may introduce a small delay to any control logic due to the need to take samples. An alternative option is to utilize an optical cell with a pump to extract material from the total product volume (Figure 4: 3d) to quantify total product yield as opposed to current production concentration. A promising advantage of this method is that we have shown precursor cleaning can be performed using the same shear exfoliation device as in the nanosheet synthesis process, suggesting that facilities costs can be minimized at industrial scales by also utilizing the same LPE and disk-centrifuge equipment for this precleaning step. Additionally, due to the universality of LPE for processing layered van der Waals materials and the batch production nature of the methodology, the facilities would not need to be constrained to a single material, although adequate cleaning to avoid material contamination would be imperative.

## Conclusion

Using MoS_2_ as a model 2D material and precursor, solvent recycling with minimal postprocessing was found to be both viable and improved production yields over existing synthesis strategies that are used in laboratories and industry today. By employing a solvent recycling scheme using liquid cascade centrifugation and EtOH recovery via distillation, we see up to 72% reduction in EtOH requirement and 82% reduction in solvent waste volume versus traditional synthesis routes, without compromising product yield over a three iteration window. EtOH recovered by distilling the waste shows minimal evidence of destabilizing impurities, indicating its reuse should be effective. On top of this, when considering the entire process, including distillation, the fresh solvent method uses 14% more energy than the recycling method. Given that mechanical exfoliation is an agnostic approach for layered van der Waals materials, this should be applicable to any polar solvent/layered material combination and to any LPE synthesis method, e.g., ultrasonication, ball milling, mixed vessel. Finally, a pathway for scale-up has been presented to support the translation of the proposed recycling concepts into industrial scale production which, with widespread implementation within industrial production of graphene and related materials could potentially save as much as ∼1B L/year of solvent and reduce solvent waste by ∼100 M L/year by 2028 based on recently published material demand forecasts.^39^

## Supporting Information Available

Extended methods and additional data(PDF)
