# Supplementary material for: Exploring Feedstock Recycling in Liquid-Phase-Exfoliated Nanosheets
Source: ACS Sustain Chem Eng. 2024 Sep 18;12(39):14363–70. doi: 10.1021/acssuschemeng.4c05845 (PMC11445721; doi:10.1021/acssuschemeng.4c05845)
Supplement: Supplementary file 1 — sc4c05845_si_002.pdf [file sc4c05845_si_002.pdf]

# Supporting Information:

# Exploring Feedstock Recycling in Liquid-Phase-Exfoliated Nanosheets

Jacob Brown and Jason Stafford*

*School of Engineering, University of Birmingham, Birmingham, B15 2TT, UK.*

E-mail: j.stafford@bham.ac.uk

| | |
|---|---|
| Pages | 9 |
| Figures | 4 |
| Tables | 0 |

# Materials & Experimental Methods

## Synthesis

Purified powders were initially distributed in 400 mL EtOH/DeI co-solvent at 30 $g\,L^{-1}$. All synthesis was performed at power setting 40 on the blender; corresponding to $\sim 11,000$ rpm to produce average shear rates of $\approx 1.8 - 2.7 \times 10^4$ $s^{-1}$ in the liquid-solid dispersion,[1] with localised shear rates in the blade passage region likely experiencing values significantly greater than this,[2] thus exceeding the binding energy of the layered $MoS_2$ precursor ($\sim 3\times10^4$ $s^{-1}$[3]). Dispersions were exfoliated for a total of 30 mins (in 1 min 'on', 2 mins 'off' cycles). A co-solvent volume ratio of 50 vol% was chosen based on work by Zhou et al.[4] 50 vol% was chosen to avoid solvent evaporation causing EtOH concentration to drop below 45 vol% as this results in a steep decline in yield. Minimal EtOH vol% change was observed in initial exfoliations and 50 vol% ratio was retained for consistency across repeats. After each exfoliation iteration EtOH concentration was measured and adjusted back to 50 vol% if necessary.

Exfoliated mixtures were left to settle for $\sim$ 24 hours to remove the bulk of the sediment before centrifugation. 24 hours was chosen to allow consistency across repeats regardless of the time of day the exfoliation was performed. In reality this could be reduced significantly, say to $\sim$ 6 hours or even less.

To check the EtOH concentration of the 90 mins exfoliated sample the synthesis was stopped every 30 mins of exfoliation time, and a small sample centrifuged at 3260 RCF to remove the majority of the sediment and check the EtOH concentration of the solvent, adjusting if necessary. The sample was then redispersed and returned to the exfoliation vessel to continue the process.

## Liquid Cascade Centrifugation

The technique involves two centrifugation steps; one to sediment and remove large, unexfoliated material, and the other to sediment the target material, also removing excess solvent and very small nanomaterial. During this process the product material is re-dispersed in fresh solvent.

For this work constant LCC bands of 1500 (Main text, figure 1a.2) and 3260 (Main text, figure 1a.3) RCF were used. Samples were centrifuged (ThermoScientific Megafuge 8) in 50 mL aliquots for 2 hours. Material was redispersed by submerging centrifuge tubes in a sonication bath (Fisherbrand Easy) with additional solvent. Sediment recovery (Main text, figure 1b.2) utilised any remaining solvent recovered from the previous stage and fresh solvent where required. Product material (Main text, figure 1a.4) was dispersed in fresh solvent each time.

# Yield vs $C_i$

A number of studies in the literature have explored the effect of the initial precursor concentration ($C_i$) with the concentration of $MoS_2$ nanosheets produced using liquid phase exfoliation.[5–7] Paton et al. observed $C_{\mathrm{MoS_2}} \propto C_i^{0.7}$ in NMP,[5] and Varrla et al. found $C_{\mathrm{MoS_2}} \propto C_i^{1.25}$ in surfactant solution.[7] Yuan et al. show that for $MoS_2$ exfoliated in 45 vol% EtOH/DeI, $C_i > 10\ \mathrm{mg\,mL^{-1}}$ returns no improvement in $C_{\mathrm{MoS_2}}$.[6] They do not quantify the relationship between $C_{\mathrm{MoS_2}}$ and $C_i$. Plotting these data sets together it seems that the Yuan et al. data will approximately fit to a power law function as with the Varrla et al. and Paton et al. data, although in the original publication this data is plotted on a non-log axis and $C_{\mathrm{MoS_2}}$ appears to plateau above $C_i \sim 10\ \mathrm{mg\,mL^{-1}}$. Interestingly both Yuan et al. and Paton et al. data give exponents $< 1$ such that increasing $C_i$ leads to diminishing returns in yield, however Varrla et al. find an exponent $> 1$ suggesting that increasing $C_i$ would progressively increase the $MoS_2$ yield which is impossible to extend beyond the limits of their experimental data

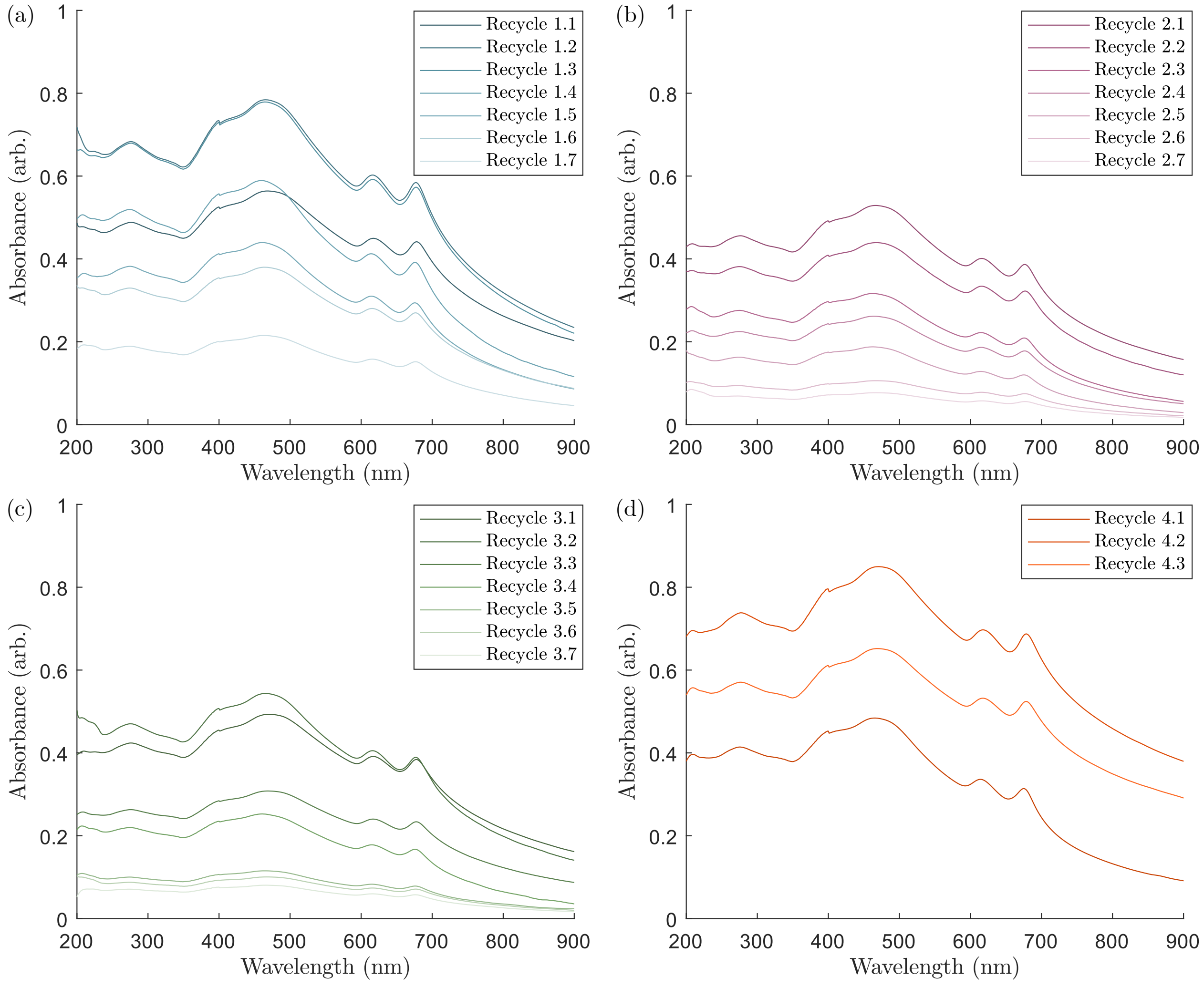


Figure S1: UV-Vis curves from the repeat data sets 1:4 (a:d)

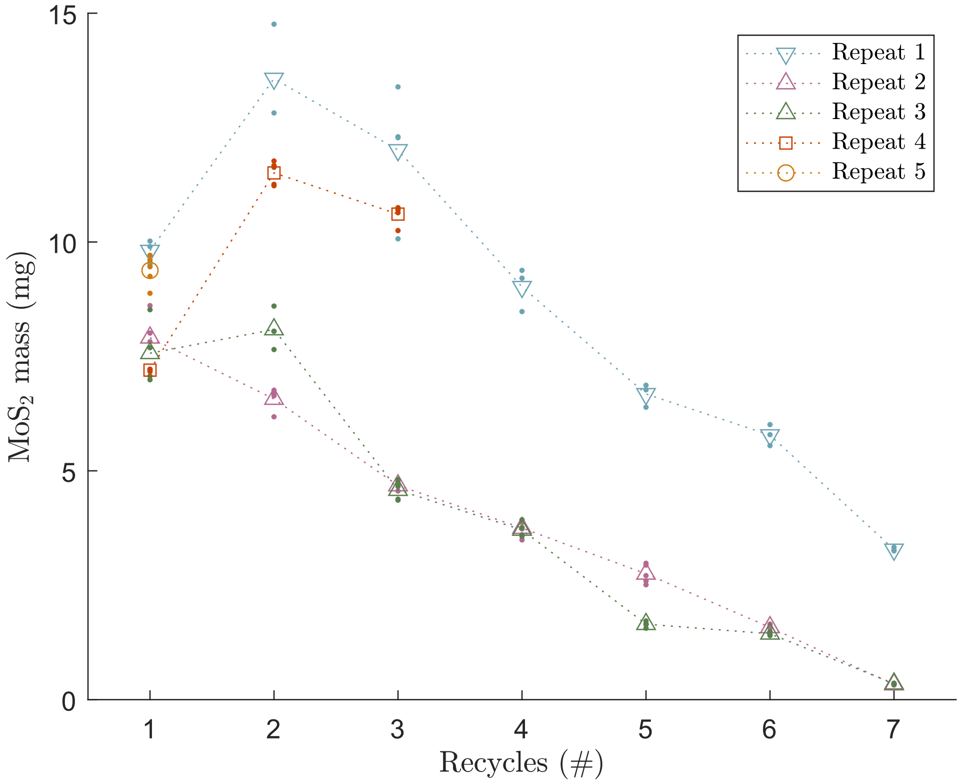


Figure S2: $MoS_2$ product masses, showing the spread in the data attributed to a lack of repeatability when removing impurities from the precursor material.

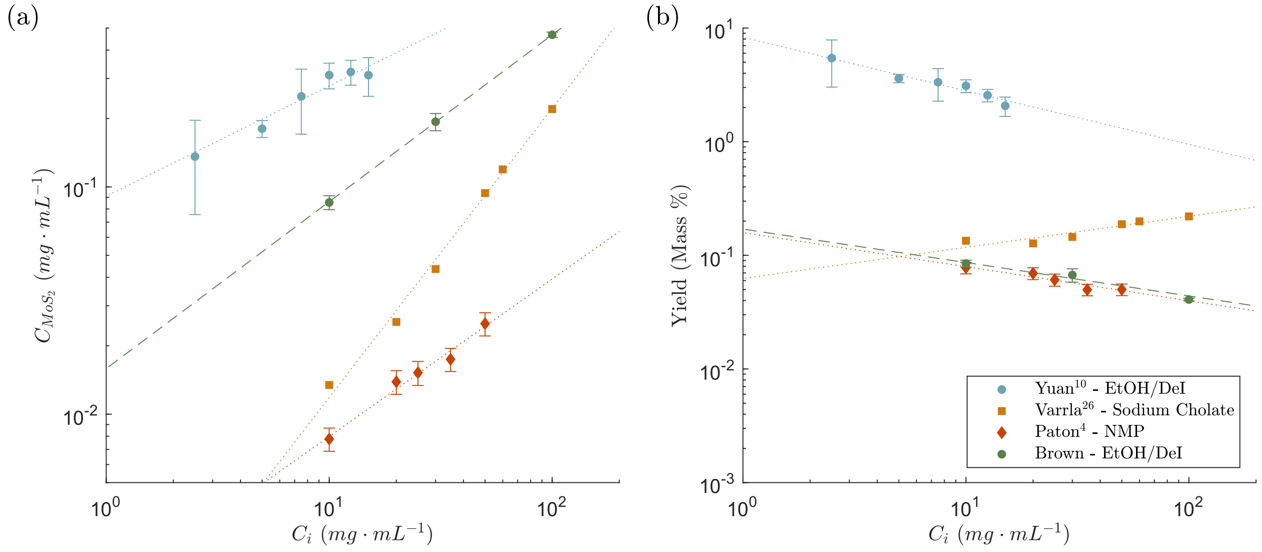


Figure S3: Graphs of initial $MoS_2$ concentration ($C_i$) against (a) product $MoS_2$ concentration and (b) product mass yield. Data extracted from works by Paton et al.,[5] Yuan et al.,[6] and Varrla et al.,[7] and plotted on a log-log axis as in Paton et al., and Varrla et al.. Green data points represent measurements taken in this work. Note: concentration data (a) for this work appears high relative to Paton et al. despite comparable yield, this is due to the concentrating effect Liquid Cascade Centrifugation has on the product material. These differences in centrifugation protocol across the different works likely result in varying product quality, this graph is simply to show the trends observed.

as this would result in yields $> 100\%$.

The above sources use a single centrifugation step to remove unexfoliated material from the dispersion before characterisation. Clearly this is not consistent with the post processing methodology used in this work. However, whilst exact values of $C_{\mathrm{MoS_2}}$ and yield will vary with post processing methodology, observed trends should be consistent. Here, $C_{\mathrm{MoS_2}}$ and yield ($Y$) are measured for $C_i = 10,\ 30,\ 100\ \mathrm{mg\,mL^{-1}}$ and follow with the data trends from both Paton et al. and Yuan et al..

# Distillation

The recovered 80 vol% EtOH UV-Vis spectra shows some evidence of impurities (Figure S4), but at a significantly reduced level compared with those in the waste solvent ($\sim 60\times$ lower), this reduction would be compounded by diluting back to 50 vol% EtOH. It is difficult to quantify the reduction due to the impurities being unidentified, and there being a clear change in the prominence of the various peaks; all three curves show peaks at $\sim 210, 238, 249$ nm as well as a broad background peak, with the $\sim 210$ nm peak being dominant in the waste solvent, and the $\sim 238$ nm peak dominant in the distillate. Normalised UV-Vis spectra can be seen in figure S4 for comparison. $MoS_2$ is marginally visible in the waste solvent curves (slight humps can be seen at $\sim 450$ and $\sim 675$ nm) but these are dwarfed by the impurities peaks and of little quantitative value.

# Optimal Distillation Point

$360 \pm 5$ mL of waste EtOH/DeI, measured at 45.51 vol%, was distilled using a rotary evaporator (BUCHI Rotovapor R-100). A total of 0.229 kWh (824.4 kJ) was used (inclusive of heating bath, rotation, vacuum pump, coolant pump) to recover $160 \pm 2$ mL of 80 vol% EtOH. This corresponds to 128 mL EtOH equivalent once diluted back to the 50 vol% co-solvent ratio, and thus $6.41\ \mathrm{kJ\,mL_{EtOH}^{-1}}$.

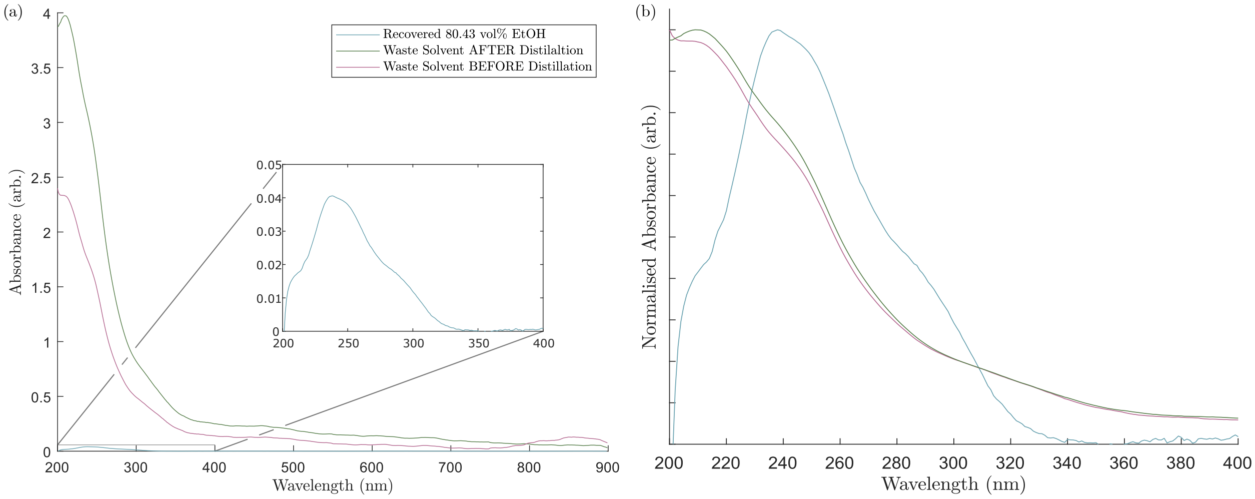


Figure S4: (a) UV-Vis curves of solvent waste before and after distillation, and the distillate. Inset shows the distillate curve in more detail. (b) Peak normalised versions of the UV-Vis curves shown in main text Figure 4 to highlight the change in the ratio of the peaks. It is clear that the prominent peak changes from ∼ 210 nm to ∼ 238 nm

By plotting product mass as a function of energy, and fitting a power law of the form $f(x) = a + bx^c$, we can offset this curve in $x$ by the energy required per synthesis (3664.8 kJ) plus the energy to distill enough solvent for one iteration ($6.41 \times 200 = 1281.2$ kJ), and in $y$ by $M_i$; i.e. plot $f(x - 3664.8 - 1281.2) + 8.12$, as in figure 3c in the main text. The intersection of these curves, ∼ 3.5 iterations, is the point at which it is energy efficient to distill the waste solvent.

# References

(1) Pérez-Álvarez, D. T.; Davies, P.; Stafford, J. Foam flows in turbulent liquid exfoliation of layered materials and implications for graphene production and inline characterisation. *Chemical Engineering Research and Design* **2022**, *177*, 245–254.

(2) Hemrajani, R. R.; Tatterson, G. B. *Handbook of Industrial Mixing*; John Wiley & Sons, Ltd, 2003; Chapter 6, pp 345–390.

(3) Biccai, S.; Barwich, S.; Boland, D.; Harvey, A.; Hanlon, D.; McEvoy, N.; Coleman, J. N.

Exfoliation of 2D materials by high shear mixing. *2D Materials* **2018**, *6*, 015008.

(4) Zhou, K.-G.; Mao, N.-N.; Wang, H.-X.; Peng, Y.; Zhang, H.-L. A Mixed-Solvent Strategy for Efficient Exfoliation of Inorganic Graphene Analogues. *Angewandte Chemie International Edition* **2011**, *50*, 10839–10842.

(5) Paton, K. R.; Varrla, E.; Backes, C.; Smith, R. J.; Khan, U.; O'Neill, A.; Boland, C.; Lotya, M.; Istrate, O. M.; King, P.; et al. Scalable production of large quantities of defect-free few-layer graphene by shear exfoliation in liquids. *Nature Materials* **2014**, *13*, 624–630.

(6) Yuan, H.; Liu, X.; Ma, L.; Gong, P.; Yang, Z.; Wang, H.; Wang, J.; Yang, S. High efficiency shear exfoliation for producing high-quality, few-layered MoS2 nanosheets in a green ethanol/water system. *RSC Advances* **2016**, *6*, 82763–82773.

(7) Varrla, E.; Backes, C.; Paton, K. R.; Harvey, A.; Gholamvand, Z.; Mccauley, J.; Coleman, J. N. Large-Scale Production of Size-Controlled MoS2 Nanosheets by Shear Exfoliation. *Chemistry of Materials* **2015**, *27*, 1129–1139.
